# Regulation of the Degradation Properties of Tyrosinase-Catalyzed Crosslinking Silk Membranes for Superficial Wound Repair

**DOI:** 10.3390/ma17122839

**Published:** 2024-06-11

**Authors:** Yu Liu, Xuping Liu, Yuhong Jiao, Mingzhong Li

**Affiliations:** National Engineering Laboratory for Modern Silk, College of Textile and Clothing Engineering, Soochow University, Suzhou 215123, China

**Keywords:** silk fibroin, self-cross-linking, degradation, tyrosinase, adjustable

## Abstract

Appropriate biodegradability to meet the demands of wound repair is critical for superficial wound repair membrane applications. Tyrosinase-catalyzed crosslinking SF (c-SF) membranes were constructed and regulated the degradation behavior in this study. The crosslinking degree of the c-SF membranes could be adjusted by reaction ratios of tyrosinase against SF (TYR/SF). Upon reaching a TYR/SF ratio of 20/6000, the degree of crosslinking increased to 88.17 ± 0.20%, without obvious changes in the crystal structure. The degradation behavior was regulated by the TYR/SF ratio and the degradation environment. All c-SF membranes remained stable after immersion without collagenase but showed an adjustable degradation behavior in the presence of collagenase. As the TYR/SF ratio increased, the residual weights increased from 23.31 ± 1.35% to 60.12 ± 0.82% after 7 days of degradation, occurring with low increased amounts of β-sheet structure and free amino acids. This work provides a new c-SF membrane with controllable rapid degradability and favorable cytocompatibility, which can help to meet requirements for biodegradable superficial wound repair membranes.

## 1. Introduction

Silk fibroin has emerged as a biopolymer with superior potential application value in tissue engineering and regenerative medicine [1], owing to its biocompatibility, processability, and relatively mild processing conditions [2,3]. Various types of silk-based materials that undergo reprocessing, modification, and modulation are used in numerous areas, such as tissue engineering [4], drug delivery [5], 3D printing [6], cell coating [7], microfluidics [8], biosensors [9], and blood vessels [10]. Nevertheless, one of the major challenges for these silk-based biomaterials is safety and efficacy in vivo tissue healing [11]. In tissue engineering and regenerative medicine, silk-based biomaterials are used as scaffolds or drug/gene carriers, which must meet the requirements of different tissues with varying degradability of the material [12]. As tissue engineering scaffolds or drug/gene carriers, silk-based materials must meet the degradability requirements for various tissue regeneration. Excessive degradation speed can aggravate structural instability or compromised mechanical properties during tissue repair, while overly slow degradation can hinder tissue adaptation and fusion with silk fibroin, thereby hindering the process of tissue regeneration and recovery [13,14]. In the field of drug/gene delivery, degradability is also an important factor affecting performance and longevity in vivo. Incompatibility between silk fibroin degradation and drug release may hinder therapeutic efficacy [15].

The degradation properties of materials are shaped by numerous factors, ranging from their intrinsic chemical composition (molecular weight, chain structure, and polymer crosslinking) [16] and physical properties (density, porosity, and water absorption) [17] to external environmental factors (pH, temperature, and enzymes) [18,19]. When the material is implanted into the tissues as a foreign body, it triggers an immune response that leads to an inflammatory response and the release of cytokines, which also accelerate the degradation of the material [20,21]. As the in vivo degradation of materials is complicated and important, regulating the degradation properties is crucial for the clinical development of silk-based biomaterials. Nevertheless, most research on silk-based biomaterials focuses on those requiring longer degradation times. In contrast, few studies on modulating the degradation of superficial wound repair membranes require shorter degradation times.

In addition to the good biocompatibility of the material [22,23], biomedical superficial wound repair membranes require relatively rapid and suitable degradability to facilitate the wound repair process [24]. For instance, because the secretions produced by postoperative nasal irritation impair recovery, superficial wound repair membranes used for intranasal stops must be degraded within 7 to 14 days to meet the demands of wound healing [25]. Although the degradation rate may vary depending on membrane material and individual differences, a superficial wound repair film for the treatment of recurrent oral ulcers typically requires a relatively rapid rate of degradation to facilitate healing of the ulcerated surface [26].

Therefore, this work aims to provide a new c-SF membrane with controllable degradability and good cytocompatibility for superficial wound repair. Regulating the degradation properties of SF-based materials to help meet requirements for biodegradable superficial wound repair membrane. Tyrosinase is a metalloenzyme that contains copper, is commonly found in microorganisms, plants, animals, and humans, and is directly connected to pigment synthesis in the body [27,28]. The tyrosinase catalyzes a reaction between tyrosine and free amino groups, which results in protein crosslinking [29,30]. Compared with chemical crosslinking reagents, tyrosinase is nontoxic and can contribute better biocompatibility to the crosslinked material [31]. As the SF contains tyrosine residues of 10–12 mol% and a certain amount of free amino groups [32,33], tyrosinase was used to produce tyrosinase-catalyzed crosslinking SF (c-SF) membranes in this study. The degradation in vitro was regulated to maintain the cytocompatibility and physical properties of the c-SF membrane, which is conducive to improving manufacturing and meeting the requirements of a biodegradable superficial wound repair membrane. 

## 2. Materials and Methods

### 2.1. Materials

*Bombyx mori* raw silk fibers were purchased from Zhejiang the Second Silk Co., Ltd. (Huzhou, China). Dulbecco’s modified eagle’s medium (DMEM), tyrosinase (T3824-25KU), fetal bovine serum (FBS), bovine serum albumin (BSA), Collagenase IA (C0130), and cell counting kit-8 (CCK-8) were purchased from Sigma-Aldrich (Darmstadt, Germany). Coomassie brilliant blue dye was purchased from Honda Biotechnology Co., Ltd. (Beijing, China). L929 cells were provided by the School of Basic Medical Sciences, Soochow University (Suzhou, China). All other chemicals were analytical grade and purchased from Sinopharm Chemical Reagent Co., Ltd. (Shanghai, China).

### 2.2. Preparation of c-SF Membrane

The fabrication of tyrosinase-catalyzed crosslinking SF (c-SF) membrane is as follows. Briefly, *Bombyx mori* fibers were degummed in a solution containing 0.6 g/L Na_2_CO_3_ at 100 ± 2 °C for 30 min, repeated three times, and then dissolved in the CaCl_2_·CH_3_CH_2_OH·H_2_O solution (molar ratio 1:2:8) for 1 h at 72 ± 2 °C with stirring. The SF solution was obtained after dialysis with a cellulose tube (MWCO 9~12 kDa) for 96 h to remove the excess salts. Then, the tyrosinase (TYR) powder was incorporated into the SF solution, resulting in the final reaction ratios of tyrosinase against SF (TYR/SF) of 0/6000, 1/6000, 2/6000, 3/6000, 5/6000, 10/6000, and 20/6000 in the mixed solution, respectively. Subsequently, the reaction was then carried out by mixing the solution at 25 ± 2 °C for 90 min. Finally, the mixed solution was poured into a dish and further dried to obtain a c-SF membrane with different reaction ratios of TYR/SF.

### 2.3. Evaluation of Crosslinking Degree

Qualitative evaluation of the tyrosine-catalyzed crosslinking reaction of the c-SF membrane was performed using Fourier transform infrared spectroscopy (FT-IR). The dried c-SF membrane was cut into microparticles with diameters of less than 40 μm, and the samples were processed into KBr pellets. FTIR was performed using a Nicolet 5700 spectrometer (Thermo Scientific, Waltham, MA, USA) in the spectral range of 400~4000 cm^−1^ and analyzed using Opus 5.0 software (Bruker, Germany). The degree of crosslinking of the c-SF membranes was determined using the ninhydrin method described in previous literature [34]. Briefly, 0.05 g of the c-SF membrane was dissolved in distilled water (bath ratio 1:30) after 1 h of equilibration. Then, the solution was mixed with 450 μL of 0.1% (*w*/*v*) aqueous ninhydrin solution and stirred slowly at 100 ± 2 °C for 20 min. After centrifugation, the absorbance of the supernatant was determined at 570 nm using an ultraviolet spectrophotometer (Hitachi, Tokyo, Japan, UV-3010). Glycine solutions of various known concentrations were used as standards. The degree of crosslinking was expressed as a percentage of the converted free amine number relative to the initial free amine number.

The tyrosine content of the c-SF membrane was determined using the high-speed amino acid analyzer (Hitachi, Tokyo, Japan, L-8900). According to the method described in previous literature [35], the c-SF membranes with TYR/SF ratios of 0/6000, 2/6000, 3/6000, and 20/6000 were hydrolyzed in 6 M HCl solution at 110 ± 1 °C under vacuum for 24 h and then reacted with ninhydrin solution. The amino acid analyzer was used to determine the identity and quantity of amino acids in the hydrolyzed products of c-SF membranes. The quantitative amino acid composition, expressed as mol% for each amino acid, was determined by external standard calibration (Amino Acid Standard H, Pierce). The samples were analyzed in duplicate (error: ±2%).

### 2.4. Physical Properties of c-SF Membrane

The secondary structures of the c-SF membranes were analyzed by XRD (X’Pert-Pro MPD, PANalytical B.V. Hollands). X-ray diffraction intensity curves were obtained at a scanning angle of 5–50° and a scanning rate of 1°/min. The c-SF membranes were cut into strips of 250 mm × 100 mm to test the mechanical properties. Tensile tests were performed using the mechanical testing machine (Instron 5567, Norwood, MA, USA) at a strain rate of 25 mm/min [36]. The elongation at break was derived from the stress–strain curve. Data for each sample were determined by averaging five tests.

### 2.5. Cells Growth and Metabolism Assay on c-SF Membrane

The viability of cells on the c-SF membranes was investigated using a cell counting kit-8 (CCK-8) [37]. L929 cells were seeded onto the c-SF membranes at a density of 5 × 10^4^ cells/well and incubated at 37 °C in a 5% CO_2_ atmosphere. According to the manufacturer’s instructions, the test was performed after 1, 3, 5, 7, and 9 days. The absorbances of each sample were measured at 450 nm using a microplate reader (Bio-Tek Synergy HT, Winooski, VT, USA). Cells seeded directly on the culture plates were used as controls.

The protein synthesized by the cells growing on the c-SF membrane was measured using the Coomassie brilliant blue assay [38]. Briefly, a standard calibration curve was constructed using BSA as the standard solution. L929 cells were seeded on c-SF membranes for 4, 6, and 8 days. After trypsinizing and centrifuging, 1.2 mL of NaOH (0.75 mol/L) was added to each sample and reacted at 100 °C for 3 min. Then, 0.1 mL of lysate was mixed with 5 mL of Coomassie brilliant blue dye and reacted for 10 min. The mixed medium was analyzed at 605 nm using a spectrophotometer. Coomassie brilliant blue dye and 100 μL cell lysates were used as control.

### 2.6. Secretion of Collagenase in L929 Cell Culture Medium

The secretion of collagenase in L929 cells was determined by ELISA kits. A standard curve of collagenase was constructed. After 3 days of culture, the supernatant of the well-grown L929 cells was selected and dripped into ELISA kits. The absorbance values of collagenase were measured at 450 nm using a spectrophotometer to calculate the amount of collagenase secreted by the cells.

### 2.7. Residual Mass of c-SF Membranes after Degradation

The c-SF membranes were cut into 3 × 1 cm squares, weighed, and immersed in a 0.05 M PBS solution (pH = 7.4, bath ratio 1:50) containing Collagenase IA (0.1 U/mL) or not (0 U/mL) at 37 ± 1 °C with slow shaking. The degradation solution was replaced with a fresh enzyme solution every day. After 1, 2, 3, 4, 5, 6, and 7 d, the degradation products and residues were collected, rinsed with deionized water, and then dried at 105 ± 1 °C to a constant weight. Quantitative changes are expressed as percentages of the remaining weight relative to the initial dry weight.

### 2.8. The Analyses of the Residues after Degradation

The surface morphology of the c-SF membrane after 0 and 6 days of degradation was examined using scanning electron microscopy (SEM, S-4800, Hitachi, Tokyo, Japan, Japan), and the structural changes were analyzed using FTIR and XRD. The secondary structure content of the c-SF membranes was measured by FTIR [39]. Briefly, Fourier self-deconvolution of the amide I region (1595–1705 cm^−1^) was performed using Opus 6.5 software (Bruker, Bremen, Germany), and the Fourier self-deconvolution spectra were curve-fitted to measure the relative areas of the amide I region components. Furthermore, the composition and content of free amino acids in the collagenase solution after degradation of the c-SF membrane were determined using a high-speed amino acid analyzer (Hitachi, Tokyo, Japan, L-8900) [40].

### 2.9. Statistical Analysis

Unless otherwise indicated, the quantitative results were acquired from triplicate samples. The data were recorded as means ± standard deviation (SD) and analyzed by SPSS (version 11.5, SPSS Inc., Chicago, IL, USA). Differences at *p* < 0.05 were considered to be significant, specifically.

## 3. Results

### 3.1. Crosslinking of the c-SF Membrane

**Figure 1:**
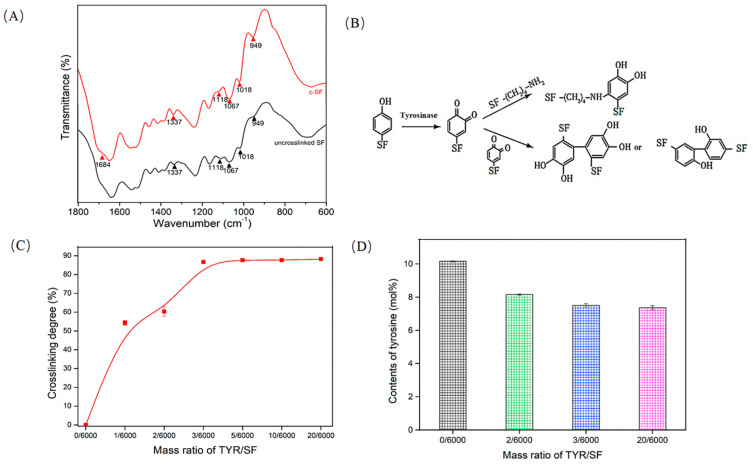
FTIR spectra (**A**) and reaction mechanism of the tyrosinase--catalyzed crosslinking SF (**B**); Effect of TYR/SF ratios on crosslinking degree (**C**) and tyrosine content of c-SF membranes (**D**).

The FTIR spectra (A) and schematic diagram (B) in Figure 1 were obtained to determine the tyrosinase-catalyzed crosslinking reaction of the c-SF membranes. Compared with un-cross-linked SF (TYR/SF = 0/6000), the intensity of the adsorption band at 949 cm^−1^, which represents the ring bending vibrations of tyrosine, was increased in c-SF membranes, indicating that the tyrosine residues in SF were oxidized by tyrosinase to form *O*-quinone residues. Furthermore, the intensity of the bands was 1337, 1067, and 1018 cm^−1^, which represents that the stretching vibration of C-C on amino side chains increased, owing to the production of methidquinone in c-SF membranes [41]. Additional small intensity changes were detected, consistent with the weak bands at 1118 cm^−1^, due to the reaction of -NH_2_ with *O*-quinone residues on the SF. The FTIR results show that tyrosinase oxidized tyrosine residues in SF to form *O*-quinones and oxidation products, which subsequently led to a reaction with amino groups of SF molecules, providing evidence for the formation of the c-SF membrane. The reaction mechanism is shown in Figure 1B.

The degree of crosslinking reflects the extent of the reaction between the free amino side chains of SF and the tyrosine residues oxidized by tyrosinase. The results in Figure 1C show that the crosslinking degree of the c-SF membranes was 54.27 ± 1.35% when the reaction ratio of TYR/SF was 1/6000. Increasing the reaction ratio of TYR/SF from 2/6000 to 3/6000 resulted in a significant enhancement in the crosslinking degree, rising from 60.33 ± 2.49% to 86.63 ± 0.81%. In addition, the increase in the degree of crosslinking became slow and stable, reaching 88.17 ± 0.20% when the TYR/SF ratio increased to 20/6000. It was mainly due to the decrease in the free amino groups of SF in the reaction process. The crosslinking degree of the c-SF membranes could be adjusted by the reaction ratios of TYR/SF. The tyrosine residues in SF were oxidized by tyrosinase to form the *O*-quinone residues during the formation of c-SF membranes, so the degree of crosslinking can be determined by changes in tyrosine content. The Tyr content of the c-SF membranes with a TYR/SF ratio of 0/6000 was about 10.16 ± 0.02 mol% but decreased to 8.16 ± 0.04 mol% as the TYR/SF ratio increased to 2/6000. The Tyr content finally stabilized at 7.35 ± 0.12 mol% when the TYR/SF ratio reached 20/6000. The probability of tyrosine undergoing catalytic oxidation increases with the addition of tyrosinase. The involvement of more tyrosine in the crosslinking reaction results in a reduction in the quantity of tyrosine present in the c-SF membranes. This result further proved that the crosslinking reaction occurred, and Tyr was the target of the tyrosinase-catalyzed crosslinking reaction system (Figure 1D).

### 3.2. Characterization of c-SF Membranes

**Figure 2:**
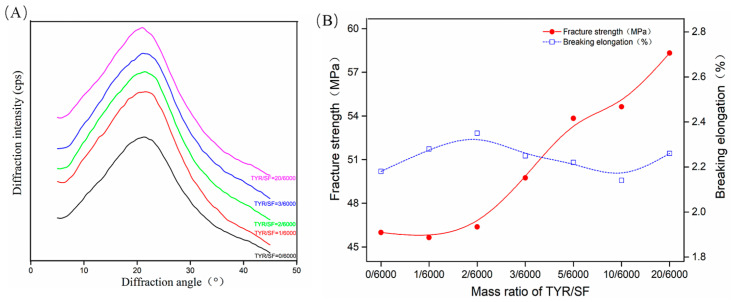
XRD curves (**A**) and mechanical properties (**B**) of c-SF membranes with different TYR/SF ratios.

The crystal structures of the SF membranes after crosslinking are shown in Figure 2A. At the TYR/SF ratio of 0/6000, only a strong diffraction peak near 22° was observed for the c-SF membrane, indicating that the condensed structure of the un-cross-linked c-SF membrane was mainly amorphous. As the TYR/SF ratio increased, no new diffraction peaks were observed, indicating that the crystal structure remained unaltered. Tyrosinase-catalyzed crosslinking did not promote regularization of the crystal structure obviously in the c-SF membrane. The c-SF membranes with TYR/SF ratios of 0/6000 were brittle and exhibited low fracture strength and elongation at break. With an increase in the TYR/SF ratio, the fracture strength of the c-SF membranes increased. However, the breaking elongation showed fluctuations, which initially increased and then decreased. The formation of the *o*-benzoquinone structure was catalyzed by tyrosinase in c-SF membranes, which then undergoes a crosslinking reaction with the amino groups in the SF molecule, accompanied by the self-polymerization of quinones. In Figure 2B, the fracture strength of the c-SF membranes with a TYR/SF ratio of 0/6000 is 45.99 ± 2.12 MPa, while the breaking elongation is 2.18 ± 0.15%. With a small addition of tyrosinase, the breaking strength of the c-SF membrane was only slightly improved. However, when the TYR/SF mass ratio was increased to 20/6000, the fracture strength reached the maximum value of 58.32 ± 4.34%. Subsequently, the mass ratio of TYR/SF further increased, leading to a decrease in the final strength of c-SF membranes. However, the observed trend in breaking elongation remained relatively constant. The primary reason for this phenomenon is tyrosinase catalyzes the formation of the o-benzoquinone structure from the tyrosine residues of the silk fibroin protein. Simultaneously with the crosslinking reaction with amino groups in silk fibroin molecules, quinones also undergo a self-polymerization reaction with each other. The addition of tyrosinase led to an increase in the interaction between SF molecules, a rise in the degree of crosslinking of the c-SF membrane, and an improvement in fracture strength. However, when the increase in tyrosinase reaches a certain extent, the molecular strength of the SF increases simultaneously with the unit length of the molecular chain in the rigid chain. Instead of hindering the movement of the molecular chain, the fracture changes from the original toughness to a brittle fracture, resulting in a decrease in the fracture strength and a loss of flexibility of the c-SF membrane.

### 3.3. Cell Viability and Collagenase Secretion on c-SF Membrane

**Figure 3:**
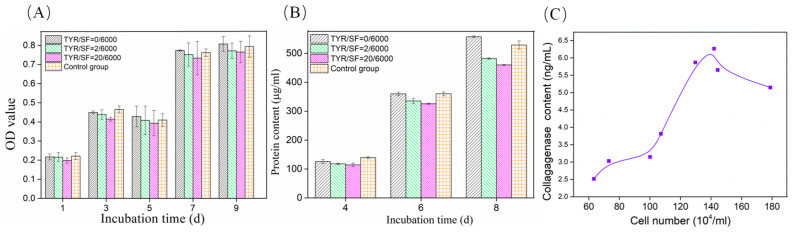
Cell viability (**A**), the total amount of protein (**B**), and collagenase I content (**C**), synthesized after L929 cells were cultured on the c-SF membrane of different TYR/SF ratios.

The cytocompatibility of the c-SF membranes was evaluated by assessing the growth and viability of L929 cells in vitro. The seeded cells were able to attach, grow, and proliferate on the surfaces of all c-SF membranes after culture. A well-adherent and spread-out phenotype in the form of antennae or scale triangles was observed on all the c-SF membranes. Mitochondrial succinate dehydrogenase in viable cells reacts with CCK-8, which can be used to assess cell growth and proliferation viability. As shown in Figure 3A, the OD values of the cells gradually increased after 1~9 days of culture, indicating that all c-SF membranes could support cell growth. The metabolic activity of the cells was assessed by measuring the total protein content synthesized by growing L929 cells on c-SF membranes (Figure 3B). After 4 days of incubation, there was no significant difference in protein content between c-SF membranes of different TYR/SF ratios. However, as culture time increased, the protein content significantly increased, indicating that all c-SF membranes were capable of supporting cell growth and metabolism. These results demonstrate that the c-SF membranes were biocompatible and maintained the metabolic activity of the cells, which is consistent with the CCK-8 assay results. Collagenase catalyzes the hydrolysis of collagen into free amino acids, thereby promoting renewal and repair of tissue. Collagenase is mainly found in collagen-rich tissues, such as skin, tendons, ligaments, and bones. Fibroblasts are the main collagen-synthesizing cells that synthesize and secrete collagenases. The secretion of collagenase in the culture medium of L929 cells was detected by ELISA. It showed that collagenase content increased with increasing cell number (Figure 3C), suggesting that the cells secreted collagenase during the growth process on c-SF membranes. However, when the number of cells exceeded 142.1 ± 1.5 × 10^4^/mL, the amount of collagenase secreted by the cells exhibited a slight decline. This phenomenon may be attributed to the fact that when the cell density reaches a certain threshold, the availability of nutrients and oxygen to each cell is relatively diminished, while the accumulation of cellular metabolites increases. This in turn affects the production of collagenase secretion. In addition, high cell density can lead to changes in cell morphology, including cell flattening and stacking. These morphological changes can affect the normal function and growth of cells, which in turn affects collagenase secretion. Collagenase was selected to study the degradation behavior of the c-SF membrane in vitro for predicting degradation behavior in vivo subsequently.

### 3.4. Degradation of c-SF Membrane In Vitro

**Figure 4:**
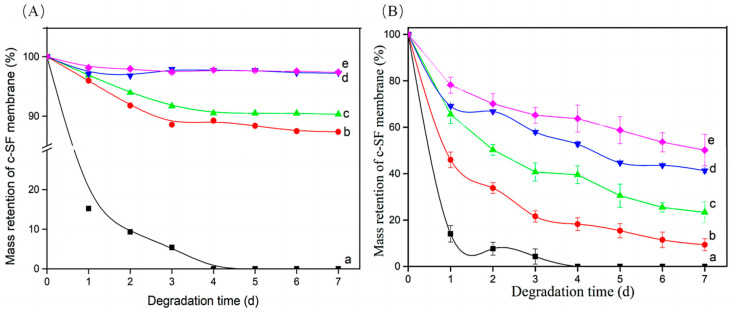
Quantitative changes in the c-SF membrane with TYR/SF ratios of 0/6000 (a), 1/6000 (b), 2/6000 (c), 3/6000 (d), and 20/6000 (e) during degradation in collagenase IA solution of 0 U/mL (**A**) and 0.1 U/mL (**B**), respectively.

The degradation behavior of crosslinked materials requires considering multiple factors, including crosslinking degree and degradation environment. Figure 4 shows the mass retention of c-SF membranes during degradation in Collagenase IA solution of 0 U/mL (A) and 0.1 U/mL (B). As shown in Figure 4A, after immersion for 1 d, the residual mass of the pure c-SF membrane (TYR/SF = 0/6000, a) was only 15.20 ± 1.51%. When the TYR/SF ratio increased to 1/6000, the residual mass of the c-SF membrane reached 95.96 ± 2.35%. When the TYR/SF mass ratio was further increased to 20/6000, the residual mass increased to approximately 98.13 ± 3.42% (A, e). After 3 days of degradation, the pure c-SF membrane completely dissolved (A, a), but the residual mass of the other c-SF membranes was above 88.56 ± 3.46%. Furthermore, all c-SF membranes remained stable in PBS solution in the absence of collagenase IA. There was only a slight reduction in the residual mass from day 3 to day 7, which may be attributed to the dissolution of some un-cross-linked SF in the buffer. After 7 d of immersion, the residual mass of the other c-SF membranes was greater than 87.35 ± 1.54% (A, b).

After 1 d of degradation in Collagenase IA solution of 0.1 U/mL (Figure 4B), the residual mass of the c-SF membrane (TYR/SF = 0/6000) was only 14.02 ± 0.57% (B, a). However, the residual masses of the c-SF membrane (TYR/SF = 2/6000) and c-SF membrane (TYR/SF = 20/6000) were 65.56 ± 1.92% (B, b) and 78.13 ± 0.42% (B, e), respectively. The mass of the c-SF membrane (TYR/SF = 0/6000) decreased significantly to 4.25 ± 1.24% after 3 days. The residual weights of the c-SF membrane decreased to 42.68 ± 1.97% (TYR/SF = 2/6000, c) and 69.36 ± 0.35% (TYR/SF = 20/6000, e). After further degradation, the c-SF membrane almost completely dissolved and degraded within 7 days (a). The difference in residual weights between the c-SF membranes increased significantly, resulting in residual weights of 23.31 ± 1.35% (TYR/SF = 1/6000, b) and 60.12 ± 0.82% (TYR/SF = 20/6000, e) after 7 days of degradation. The degradation rate of the c-SF membrane decreased as the reaction ratio of TYR/SF increased, indicating that the degradation behavior of the SF membrane could be tuned by the degree of tyrosinase-catalyzed crosslinking.

### 3.5. Morphological Changes during Degradation

**Figure 5:**
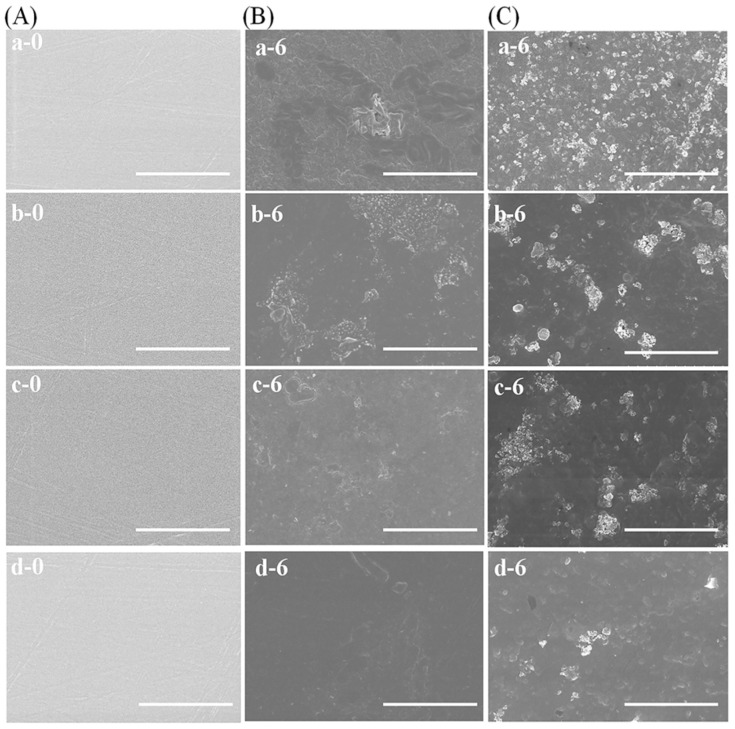
SEM images of the c-SF membrane with TYR/SF ratios of 1/6000 (a), 2/6000 (b), 3/6000 (c), and 20/6000 (d) before degradation (**A**) and during degradation for 0 and 6 days in collagenase solution of 0 U/mL (**B**) and 0.1 U/mL (**C**). Scale bars: 10 μm.

The morphology of the c-SF membrane before and after degradation in different concentrations of collagenase IA solution was examined using SEM, as shown in Figure 5. The surfaces of all the c-SF membranes were smooth before degradation (A). As illustrated in Figure 5B, the surface of the c-SF membrane became rougher after 6 days of degradation in the solution despite the absence of Collagenase I (0 U/mL). Moreover, the degree of roughness decreased as the TYR/SF ratio increased further. The c-SF membrane with a TYR/SF ratio of 20/6000 exhibited only a few spots on an otherwise flat surface (B, d-6). However, after 6 days of degradation in collagenase I solution, sheet-like structures and small pores appeared on the surface of all the c-SF membrane (C). Furthermore, the c-SF membrane with a low TYR/SF ratio of 1/6000 (C, a-6) exhibited a higher degree of surface roughness, more shrunken pores, and more sheet-like structures than the c-SF membrane with a high TYR/SF ratio of 20/6000 (C, d-6). This suggested that c-SF membranes are more susceptible to enzymatic degradation, and the degree of degradation depends on the TYR/SF reaction ratio.

### 3.6. Conformational Changes in c-SF Membrane

**Figure 6:**
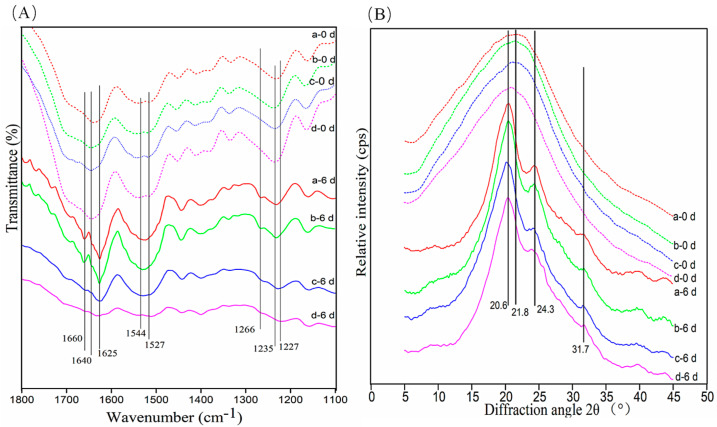
FTIR spectra (**A**) and XRD data (**B**) of c-SF membrane with TYR/SF ratios of 1/6000 (a), 2/6000 (b), 3/6000 (c), and 20/6000 (d) after enzymatic degradation for 0 and 6 days.

The conformational transformation in the c-SF membrane after degradation was analyzed by FTIR. As shown in Figure 6A, the c-SF membrane with a low TYR/SF ratio (TYR/SF = 1/6000) has obvious peaks at 1640 cm^−1^ (random coil, amide I), 1544 cm^−1^ (α-helix, amide II), and 1235 cm^−1^ (random coil) before degradation (a-0 d), which correspond to c-SF membranes with high TYR/SF ratio of 20/6000 (d-0 d). It indicated that the conformation of the c-SF membrane did not change with increasing TYR/SF reaction ratios. After 6 days of degradation, the peaks at 1640 cm^−1^ (random coil) disappeared from the observation of the c-SF membrane with a low TYR/SF ratio of 1/6000, whereas strong new peaks at 1625 cm^−1^ (β-sheet). Meanwhile, there was a shift in the peak at 1235 cm^−1^ (random coil), while peaks at 1266 cm^−1^ (α-helix) appeared (a-6 d). The same trend in structural changes was also observed in the c-SF membrane with a high TYR/SF ratio of 20/6000 after 6 days of degradation (d-6 d). Furthermore, the change in the crystal structure of the c-SF membrane was determined using XRD (Figure 6B). The c-SF membranes with TYR/SF ratios of 1/6000 (a-0 d) and 20/6000 (d-0 d) showed similar significant scattering diffraction peaks at 21.8°, indicating that the crystal structure did not obviously change with increasing reaction ratios of TYR/SF. After 6 days of degradation, the typical peaks occurred at 20.6° (Silk II), 24.3° (Silk II), and 31.7° (Silk II) (a-6 d, d-6 d). The results declare that the amorphous regions of the c-SF membrane were preferentially degraded by collagenase regardless of the different TYR/SF ratios and that the secondary structures of the residues in the c-SF membrane after degradation were mainly β-sheet structures.

### 3.7. Protein Secondary Structure Content after Degradation

**Figure 7:**
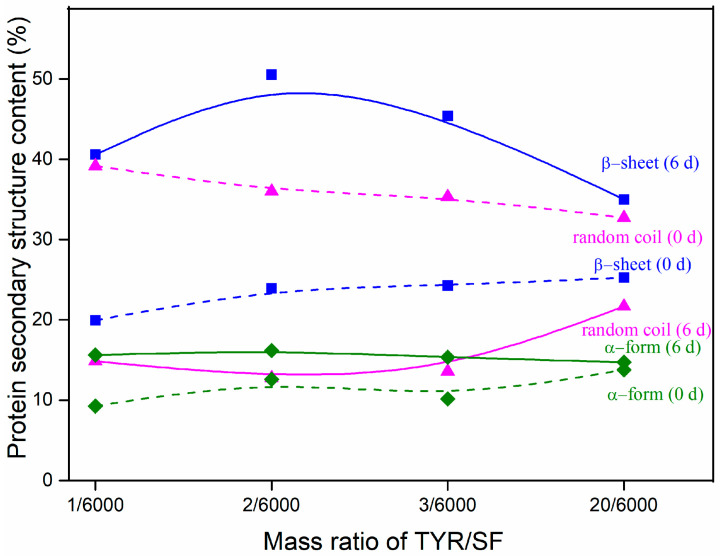
Protein secondary structure content of c-SF membrane before (0 d) and after degradation (6 d).

The changes in protein secondary structure content of the c-SF membrane before and after degradation by collagenase are shown in Figure 7. The c-SF membrane with a TYR/SF ratio of 1/6000 had a 39.17 ± 1.21% random coil structure, a 9.27 ± 1.03% α-form, and a 19.94 ± 0.34% β-sheet prior to deterioration (0 d). Nevertheless, the random coil structure dominated the secondary structure of the c-SF membrane, despite a slight increase in α-form and β-sheet structures with an increase in TYR/SF ratios. The percentage of random coil structures in the c-SF membrane with TYR/SF ratios of 1/6000 dropped to 14.87 ± 0.79% following 6 days of degradation. Meanwhile, the content of α-form and β-sheet increased to 15.64 ± 1.13% and 40.57 ± 0.71%, respectively (0 d). When the TYR/SF ratio reached 20/6000, the proportion of random coil structures reduced to 21.69 ± 0.86%, while α-form and β-sheet structures increased to 14.72 ± 1.21% and 34.98 ± 1.33%, respectively. The results indicate that α-form and β-sheet structures of c-SF membranes increased and random coil structure decreased during degradation with varying TYR/SF mass ratios. And the degree of change depended on the TYR/SF mass ratio. The random coil structure is characterized by a loose arrangement of the peptide chains and unstable bonds, which is susceptible to swelling in fluid and loosening under force and easy to contact with collagenase molecules for hydrolysis. The α-form structure is substable in comparison with the β-sheet structure, which contains a tightly packed peptide chain that is difficult to break by force. Thus, the main reason for the mass decrease resulting from the enzymatic hydrolysis of the c-SF membrane is the degradation of the poorly regular, irregular, curly structure of the protein, which improves the regularity of the residue’s cohesive structure. The proportion of α-form and β-sheet structures rises while the random coil structures fall. The lower TYR/SF ratio of the c-SF membrane results in a greater change in structural content after degradation; therefore, TYR/SF mass ratios can be used to control the degradation rate of the c-SF membrane.

### 3.8. Changes in Free Amino Acid Content after Degradation 

**Figure 8:**
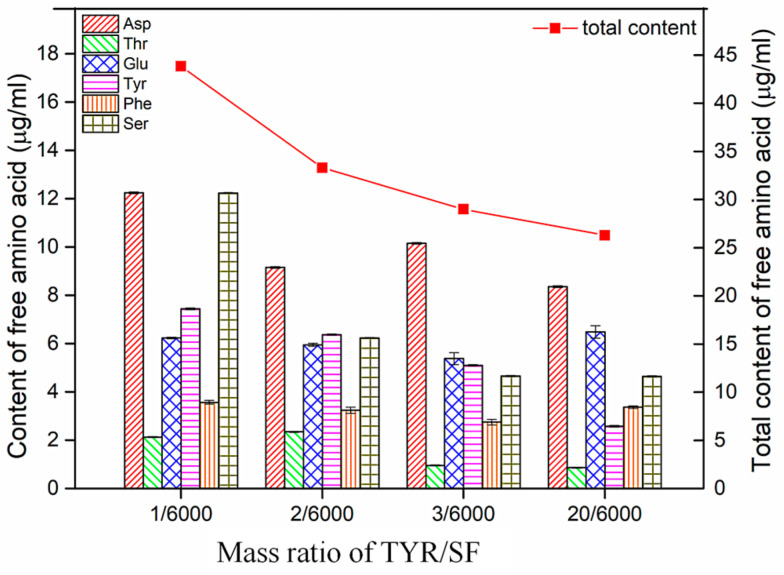
Content of free amino acid in collagenase solution after degradation of 6 d.

The effect of free amino acids from material degradation products on surrounding tissue is a complex issue that depends on various factors, such as the environmental characteristics and amino acid composition and content of tissue. In general, a low content of free amino acids does not have a negative impact on surrounding tissues and may even have a trophic effect, contributing to cell growth and repair. However, an excess of free amino acids is likely to cause an inflammatory reaction in the surrounding tissue. This is because amino acids are biologically active molecules that, when in excess, can trigger an immune system response, leading to the infiltration of inflammatory cells and tissue swelling. In addition, certain free amino acids can interfere with normal cellular metabolic pathways and impair cell proliferation, differentiation, and function. Amino acid analysis of degradation products in vitro can be helpful in evaluating the biosafety of materials in vivo. Figure 8 illustrates the free amino acid content of the c-SF membrane in a collagenase solution after degradation. After degradation by collagenase, the c-SF membrane with a TYR/SF mass ratio of 1/6000 contained several free amino acids, including aspartic acid (Asp), threonine (Thr), glutamic acid (Glu), serine (Ser), tyrosine (Tyr), and phenylalanine (Phe). The total amount of free amino acids was 43.84 ± 0.07 µg/mL. The results demonstrate that the collagenase not only degraded the c-SF membranes but also indicated that the random coil structures were the most susceptible to degradation during the process. The same amino acids were present in the degradation solution of the c-SF membrane with a TYR/SF ratio of 2/6000, but the total amount of free amino acids dropped to 33.3 ± 0.13 μg/mL. As TYR/SF mass ratios increased to 20/6000, the total amount of free amino acids decreased to 26.3 ± 0.21 μg/mL, suggesting that c-SF membranes with higher TYR/SF mass ratios were more resistant to degradation by the enzyme. These free amino acids are stable under physiological conditions and are tolerated by tissue. As major components of fibrinogen and coagulation factor VIII, Ser and Glu, in degradation products, contribute to promoting the synthesis and expression of coagulation factors around the wound. So, the degradation products of free amino acids can potentially promote wound healing. This indicates that c-SF membranes have less impact on surrounding tissue during the degradation and can be considered safe for superficial wound repair.

## 4. Summary

A self-cross-linking SF (c-SF) membrane with controllable degradation behavior was fabricated. After an environmentally friendly tyrosinase-catalyzed crosslinking reaction, the c-SF membrane maintained cytocompatibility and adjustable rapid degradability. After 7 days of collagenase degradation, the residual weights of the c-SF membrane decreased to approximately half of its original mass. The free amino acids in the degradation products have the potential to facilitate wound healing. This work provides a new c-SF membrane with controllable rapid degradability, which can help to achieve the application needs for SF-based biodegradable superficial wound repair membranes.

## Data Availability

The raw data supporting the conclusions of this article will be made available by the authors on request.
